# Gender Dysphoria in an Adolescent Presenting With Self-Initiated Hormone Therapy and Genital Self-Mutilation: A Case Report From Bangladesh

**DOI:** 10.7759/cureus.114095

**Published:** 2026-08-06

**Authors:** Shams Ibne Karim, S. M. N Huda, MD. Maminul Haque, Mohammad S Ahsan

**Affiliations:** 1 Child and Adolescent Psychiatry, Bangladesh Medical University, Dhaka, BGD; 2 Psychiatry, Bangladesh Medical University, Dhaka, BGD

**Keywords:** bangladesh, gender-affirming care, gender dysphoria (gd), self-harm, transgender youth

## Abstract

Gender dysphoria (GD) in adolescents requires timely, supervised gender-affirming care. We report a 16-year-old transgender girl in Bangladesh who, lacking access to formal services, self-initiated feminising hormone therapy with oestradiol and spironolactone and described substantial improvement over three and a half months. On psychiatric admission, the hormones were stopped because no clinician held prescribing authority for gender-affirming treatment in a minor and the provenance of her medication could not be verified; lithium, prescribed for anti-suicidal effect, was stopped the same day. She deteriorated and, on the thirteenth day, incised her scrotum in a premeditated attempt at bilateral orchiectomy requiring emergency repair, having stated that intention repeatedly. Means restriction, ligature-risk removal and family-delegated one-to-one observation had been in force since admission and remained so; the blade came from outside the hospital during a brief unsupervised interval. A modified scale for suicidal ideation the next day scored zero. These events occurred against a background of major depressive disorder diagnosed independently by three psychiatrists and suicidal ideation documented more than a year before she obtained any hormone. The case illustrates the absence of any option between unsupervised self-medication and complete cessation, and the limits of bedside means restriction on an open ward when a patient discloses a specific method.

## Introduction

Sexual health has historically been an underexplored area of medical research and clinical practice in Bangladesh. Despite substantial healthcare progress in recent decades, significant challenges remain in the recognition and treatment of psychosexual disorders. These conditions are prevalent but seldom discussed, owing to socio-cultural taboo, religious proscription, limited health literacy and low public awareness [1], although multidisciplinary training initiatives have begun to draw clinicians toward the field [2].

Gender dysphoria (GD) is clinically significant distress arising from incongruence between experienced gender and sex assigned at birth. In DSM-5-TR, the adolescent and adult diagnosis requires marked incongruence of at least six months, evidenced by at least two of six indicators, with significant distress or functional impairment [3]. Access to gender-affirming care is associated with improved mental health outcomes in transgender youth [4], and current guidance supports hormone therapy for adolescents with confirmed, persistent incongruence who can consent, within multidisciplinary assessment and monitoring [5,6]. Where such services are unavailable, adolescents may obtain hormones outside formal care, with substantial risk [7].

One distinction should be drawn at the outset. The hijra or third-gender category recognised in South Asia is sociocultural and, since the Bangladesh Government's notification of January 2014, administrative [8]. It is not a diagnostic entity: it encompasses people with gender incongruence alongside those with differences of sex development, and carries a distinct history of social exclusion [9]. Transgender people outside that category have no separate legal recognition [10]. The two categories overlap without coinciding, and the distinction acquires force when clinical pathways attach to one and not the other.

Genital self-mutilation (GSM) is a severe manifestation of unmanaged GD; a systematic review found gender dysphoric conditions to be a major comorbidity among male GSM cases, with treatment inaccessibility repeatedly cited as precipitant [11]. We describe an adolescent who self-initiated hormone therapy in the absence of formal care and performed GSM during an admission on which that therapy had been stopped.

## Case presentation

A 16-year-old adolescent assigned male at birth was admitted to the psychiatry department of a tertiary teaching hospital in Dhaka with longstanding gender incongruence, an established depressive disorder, and multiple self-harm behaviours.

Developmental and psychiatric history

From four to five years of age she consistently identified as female, preferring female peers and feminine-coded activities. Both she and her father described this as continuous through childhood and adolescence with no period of remission. Male puberty at 11 to 12 years intensified her distress, particularly over height, thyroid cartilage, shoulder breadth and jawline. She reported attraction to boys.

She met DSM-5-TR criteria for gender dysphoria in adolescents and adults [3], with all six criterion A indicators documented, criterion B met by pervasive distress and academic decline, and duration exceeding a decade. Three psychiatrists in two cities had reached this diagnosis independently over the preceding eighteen months, each also diagnosing a depressive disorder: depressive features at 14 years, unipolar depressive disorder eight months before admission, and major depressive disorder with anxious distress three days after that. The receiving centre recorded major depressive disorder with gender dysphoria two months before admission and advised hospitalisation then. All were unstructured clinical judgements against DSM-5-TR criteria; no structured interview or GD-specific instrument was used by any assessor, or during the admission. She had received escitalopram, mirtazapine, clonazepam and lithium, the last introduced eight months before admission for anti-suicidal effect rather than for any suspected bipolar disorder. Adherence was recorded as poor on one occasion.

Her self-harm history included wrist cutting, attempted ligature strangulation, breath-holding and attempted smothering. Suicidal ideation was documented at her first assessment, fourteen months before she began hormone therapy. One month before admission, she took sixteen tablets of bisoprolol 2.5 mg with suicidal intent after her family withheld her hormones.

Baseline endocrine investigation

A panel performed eighteen months before admission, at age 14 and fourteen months before she commenced oestradiol, showed luteinising hormone 6.14 mIU/mL (2.00-12.00), follicle-stimulating hormone 4.26 mIU/mL (1.00-8.00), total testosterone 4.68 nmol/L (age 12-15 interval 3.46-11.00), prolactin 10.70 ng/mL (2.10-17.70), free thyroxine 1.05 ng/dL (0.70-1.85) and thyroid-stimulating hormone 1.18 µIU/mL (0.47-5.01). All were within age- and sex-specific reference intervals, consistent with established puberty. No hormonal investigation was done during the admission, and serum oestradiol was never measured at any point in her care.

Self-initiated hormone therapy

Four months before admission, she obtained oestradiol and spironolactone without prescription, starting with oral oestradiol 2 mg and spironolactone 50 mg daily and increasing to 4 mg and 100 mg when she saw no adverse effect. After seventeen days she changed to sublingual oestradiol, explaining that she had read that this route gave different oestradiol and estrone profiles and reduced hepatic exposure; we report that as her stated rationale, its pharmacological accuracy is unassessed. Over three and a half months, she reported softer skin, reduced acne, fewer spontaneous erections and breast development she described as Tanner stage 2, with remission of depressive symptoms and cessation of self-harm urges. Her father independently described a marked reduction in self-harm over the same period, recurring after discontinuation. Both accounts are retrospective and uncorroborated, from informants with an evident stake in resumption.

Index admission

Hormone therapy was discontinued on the day of admission for two reasons: no clinician in the department held prescribing authority for gender-affirming treatment in a minor and no institutional pathway existed to seek it; and the medication, obtained without prescription, could not have its provenance or content verified without endocrinological supervision, which was unavailable. Discontinuation was abrupt, with no taper and no substitute intervention for the anticipated distress. Lithium was stopped the same day and escitalopram 10 mg each morning commenced. No standardised measure of depressive or anxiety symptoms and no GD-specific instrument was used at any point, so symptom severity is described qualitatively.

Risk of Self-Harm was Identified and Acted on From Day one

Sharps removed and access restricted, ligature-risk items placed out of bounds, the file flagged, one-to-one observation instructed and delegated to her family attendant, and nursing observations at intervals. A written undertaking acknowledging risk was obtained from the guardian. The ward ran an open general psychiatric model with family attendants at the bedside; dedicated adolescent psychiatric nursing and staff-delivered graded observation were unavailable.

She became withdrawn, tearful, sleep-disturbed and minimally communicative, and restricted food intake to achieve a slimmer physique. On day four she was found collapsed in the ward bathroom at about 19:30, having, according to her father, felt dizzy beforehand; no medical assessment of that episode is documented, since no observations, examination or investigations appear in the record. We report the gap rather than omit the event. On day seven she was absent from her bed at the morning round. She repeatedly said she would cut off her genitals if hormones were not reinstated at discharge. No endocrinology, adolescent medicine or clinical ethics consultation was sought before the index event, none being available, and urology was not involved until the day of injury.

Genital self-mutilation

On day 13 at about 13:30 she incised the scrotum with a blade in the ward bathroom, exposing the right testis. The day one precautions remained in force; the blade had come from outside the hospital during a brief unsupervised interval. Under local anaesthesia, exploration showed cut injuries running in variable directions in the right scrotal region with the testis exposed and the spermatic cord intact. The wound was irrigated with normal saline, haemostasis secured with diathermy and closure performed with 4-0 polyglactin; tetanus toxoid and intravenous ceftriaxone were given. Olanzapine 10 mg nocte was added that evening for rapid tranquillisation of postoperative restlessness and continued to discharge. It was not prescribed for a suspected psychotic process; mental state examination before and after the event recorded thought and perception as normal throughout. She explained that she had intended bilateral orchiectomy to suppress testosterone production, and had chosen the hospital so that surgical care would be immediately available. A modified scale for suicidal ideation [12], administered on day 14 in screen-and-stop form, scored 0.

In a written account prepared during the admission, she distinguished the distress of dysphoria from the relief of recognising herself, and asked for hormone therapy under an experienced endocrinologist, gradual recognition as female by her family and community, and surgery only after three or four years of hormone therapy.

She was discharged on day 18 with diagnoses of gender dysphoria and major depressive disorder, euthymic and with the wound healed, on escitalopram 10 mg and olanzapine 10 mg.

Follow-up

She resumed her previous self-administered regimen within days of discharge. Review was by telephone at two weeks and face-to-face with a family informant at one month; both were unstructured clinical assessments and no instrument was administered. Screening investigations requested two months before admission were completed at two weeks, by which point she had taken spironolactone 100 mg daily for about ten days: potassium 4.2 mmol/L (3.5-5.1), sodium 147.0 mmol/L (136-145), creatinine <1.0 mg/dL (0.7-1.3), alanine aminotransferase 36 U/L (10-45), thyroid-stimulating hormone 0.89 µIU/mL (0.25-5.00), and a normal electrocardiogram with QTc 420 ms. Haemoglobin was 12.2 g/dL (13.5-17.5) with microcytic hypochromic indices and red cell distribution width 16.7%, consistent with iron deficiency and possibly reflecting the food restriction or operative blood loss. At one month, the injury had healed without residual complication, and dysphoria remained severe. Escitalopram and olanzapine continued; lithium was not reinstated. Four days after the index event, while she was still an inpatient, her family sought the help of a practitioner of ruqyah on her behalf.

The chronological sequence of events is summarised in Table 1.

**Table 1:** Chronological timeline of clinical events in a 16-year-old transgender adolescent with gender dysphoria presenting with self-initiated hormone therapy and genital self-mutilation. LH, luteinising hormone; FSH, follicle-stimulating hormone; FT4, free thyroxine; TSH, thyroid-stimulating hormone.

Time point	Event
Age 4–5 y	Persistent female identification; feminine-coded activities and female peers
Age 11–12 y	Male puberty; dysphoria intensifies over masculine secondary sex characteristics
18 months before (age 14)	Discloses transgender identity to father; repeated self-harm. First assessment: GD with depressive features; suicidal ideation documented; endocrine panel normal for age
8 months before	Second and third assessments: GD with depressive disorder; lithium started for anti-suicidal effect
4 months before	Self-initiated oestradiol 2 mg + spironolactone 50 mg daily, later 4 mg + 100 mg; sublingual after 17 days
Over 3.5 months of use	Softer skin, reduced acne, self-staged Tanner 2 breast development; depressive symptoms remit, self-harm urges cease (retrospective informant report)
2 months before	Receiving centre records MDD with GD; admission advised
1 month before	Overdose of 16 tablets bisoprolol 2.5 mg after family withheld hormones
Day 1	Admission. Hormones and lithium stopped, escitalopram started. Sharps and ligature restriction, file flagged, family-delegated 1:1 observation
Days 1–12	Deterioration: withdrawal, tearfulness, sleep disturbance, food restriction, repeated intent to self-mutilate; precautions unchanged; no specialist input available
Day 4	Bathroom collapse, no assessment documented
Day 7	Absent from bed at morning round
Day 13	Scrotal incision, blade from outside the hospital; found by father; testis exposed, cord intact; repair under local anaesthesia; olanzapine added
Day 14	MSSI score 0
Day 18	Discharge, euthymic; hormones resumed within days
Follow-up at 2 weeks and 1 month	Reviews; investigations on spironolactone within reference limits; injury healed; dysphoria severe

## Discussion

This patient met DSM-5-TR criteria for gender dysphoria [3]. The act was premeditated, method-specific, and explicitly intended to suppress testosterone, which makes it more consistent with GD-related GSM than with nonspecific self-harm [11]. Several observations converge. The Modified Scale for Suicidal Ideation scored zero the following day. She chose an inpatient setting precisely so that surgical care would be available, which is incompatible with an intention to die. Her own written plan sought supervised hormone therapy first and surgery only after three or four years, closer to guideline sequencing than to impulsivity. What she attempted with a blade was the intervention she had been asking a health system to provide.

Unsupervised hormone use in adolescents carries real risk: thromboembolism, cardiovascular events, hyperkalaemia, hepatic dysfunction, and possible effects on bone density and fertility [5,6]. None had appeared on the single occasion she was tested while taking the drugs, which is one time point in one patient. The only biochemical monitoring she received during unsupervised use arrived incidentally, in a panel ordered for other reasons months earlier; that it proved reassuring was fortune rather than surveillance.

Her deterioration followed discontinuation closely, but discontinuation coincided with admission itself, separation from home, new medication and continuing family conflict, and a single case cannot disentangle these. Nor did her distress begin with hormone withdrawal: three psychiatrists had independently diagnosed a depressive disorder and treated it with three drug classes over eighteen months, and suicidal ideation was recorded fourteen months before she obtained any hormone.

One coincidence deserves note. Two drugs were stopped on the same day, one of them lithium, prescribed for anti-suicidal effect. Randomised evidence supports a reduction in completed suicide with lithium but found no significant effect on deliberate self-harm [13], the category this event falls into, so we do not suggest a causal role. The narrower point stands: an adolescent with a recent overdose lost her hormones and her anti-suicidal agent within twenty-four hours. Medication reconciliation on admission should ask what is being stopped, not only what is being started.

The admission dilemma

The choice was framed as binary: continue an unsupervised regimen of unverified provenance, or stop. Intermediate positions existed in principle, including continuation at her established dose under observation pending urgent endocrinological advice, a structured taper, or monitoring while a prescribing decision was reached. Each required prescribing authority for a minor, formulary access, or a consultant willing to advise on a gender-affirming indication, and none existed. The question is not whether the admitting clinicians erred within the options available to them, but why the option set was so narrow.

Means, perimeter and the limits of delegated observation

The precautions that failed here were not absent ones. Risk was identified on day one and acted on; the measures were proportionate, they were instituted before rather than after she disclosed a specific plan, and they remained in force on the day of the act. The event happened anyway, because the blade came from outside the hospital during a brief unsupervised interval.

Two vulnerabilities follow, and neither is remedied by doing more of what was already being done. The first concerns perimeter rather than possession. Means restriction on an open ward controls what a patient holds, not what a patient can obtain. Where attendants and visitors move freely, and patients are not confined, a determined and method-specific intention will find a route around any restriction applied at the bedside; the question is not whether the locker was searched but whether anything crosses the threshold unchecked.

The second concerns the observation model. One-to-one observation delegated to a family attendant is the only form available in most wards of this kind, and it is a genuine resource that should not be dismissed. It is not equivalent to staff-delivered continuous observation. The attendant is untrained, emotionally involved, cannot be relieved on a roster, and was drawn from the family whose confiscation of her medication had precipitated an earlier overdose. The gaps show in this record: an unassessed collapse on day four, an absence from her bed on day seven, an unsupervised interval on day 13.

When a patient on an open ward discloses a specific method, bedside means restriction should therefore be treated as necessary but not sufficient. The questions that follow are whether the ward can control its perimeter and sustain observation at the intensity the disclosure warrants. Where it cannot, that limitation belongs in the risk formulation and should be shared with the family, and should weigh in decisions about the length of admission and whether it is achieving anything the patient could not at home.

Instrument choice is a separate lesson. The Modified Scale for Suicidal Ideation scored zero the day after a near-castration, and would have scored the same when she first disclosed her intention. Suicidal ideation scales measure what they were built to measure; they do not detect a method-specific, non-suicidal, gender-motivated intention to remove an organ. A low score on such an instrument is not evidence that a plan of this kind has abated.

Family, help-seeking and socio-legal context

Family non-acceptance likely amplified her distress. Family acceptance is associated with better outcomes among lesbian, gay, bisexual, and transgender (LGBT) young people [14], whereas repeated suppression here appeared to worsen risk. The family's recourse to religious healing during the admission is better read as help-seeking under an explanatory vacuum than as obstruction, and it is not unusual: in a Bangladeshi pathways study only 27.5% of patients reached a psychiatric provider at first contact and 30% consulted a non-medical provider first, most often a traditional or religious healer, with a median interval to psychiatric care of one year [15]. Clinicians should expect parallel help-seeking and ask about it.

The legal position deserves stating precisely. The Ministry of Social Welfare notification of 22 January 2014, following a Cabinet decision the previous November, identified and recognised the hijra population as hijra gender [8]. It is a single operative sentence: it defines neither the category nor the criteria for entry to it, makes no reference to differences of sex development or gender dysphoria, and provides for no medical assessment and no clinical pathway. The recognition is administrative and nominal, and its substantive content has been settled by practice instead. In January 2015 the Ministry of Health requested medical examination of hijra applicants to a government employment scheme to identify "authentic hijras"; applicants then underwent physical examination, ultrasonography and forensic assessment before being told they were men [16]. The operative criterion was anatomical, and this patient's own investigations place her outside it. Consensual same-sex activity also remains criminalised under section 377 of the Penal Code 1860 [17], which addresses acts rather than identity and does not on its face reach medical treatment but forms the background against which clinicians assess their exposure. No instrument we could identify either authorises or prohibits gender-affirming treatment for an adolescent with GD in Bangladesh, and caution in that vacuum is not irrational.

Limitations

The exposure was never biochemically characterised. No oestradiol was measured at any point, no panel was performed during the admission, and the only endocrine data predate hormone therapy by more than a year. The reported improvement rests on retrospective informant report, and breast development was staged by self-report. No depression, anxiety, or GD-specific instrument was used; the only standardised measure obtained was a suicidal ideation scale, administered after the event. The day four collapse was never medically assessed. Follow-up ran to one month by unstructured assessment. As a single uncontrolled case, this report cannot establish causation.

## Conclusions

An adolescent with gender dysphoria and a concurrent major depressive disorder improved on a regimen she obtained and administered herself, deteriorated after it was stopped on admission for reasons individually defensible and collectively leaving no alternative, and then inflicted a potentially fatal injury. These events occurred in the context of limited gender-affirming services, family pressure regarding gender expression and abrupt hormone discontinuation, and against a background of depressive illness and suicidal ideation predating any hormone use; no single factor can be identified as the cause from one case, and we do not claim otherwise. Several lessons follow independently of that question. Adolescents with GD in this setting have access to unsupervised hormones and to emergency surgery but to nothing in between, and a clinician facing an unplanned admission needs to know beforehand which intermediate options are locally achievable, because that cannot be worked out from first principles at three in the morning. Bedside means restriction was in place here from the first day and was not enough: an open ward cannot control what a patient obtains from outside it, and family-delegated observation cannot be sustained at the intensity a disclosed plan warrants, so wards operating this model should name that limitation in the risk formulation rather than treat bedside restriction as closure. Parallel help-seeking outside the medical framework should be anticipated and asked about. The 2014 recognition is an administrative status with no clinical content attached, so what this case locates is not a pathway needing extension but the absence of one. Whether building it reduces severe self-harm is a question for prospective study, and we offer this report as grounds for asking it.

## References

[1] Maruf MM, Mubassara L, Ahsan MS (2024). Psychosexual disorders in Bangladesh: from bench to community. Mental Health in Bangladesh.

[2] Ahsan MS, Mubassara L, Islam AK (2025). South Asian Society for Sexual Medicine School, Bangladesh: a multidisciplinary training initiative in an under-resourced country. Sex Med.

[3] American Psychiatric Association (2022). Diagnostic and statistical manual of mental disorders.

[4] Tordoff DM, Wanta JW, Collin A, Stepney C, Inwards-Breland DJ, Ahrens K (2022). Mental health outcomes in transgender and nonbinary youths receiving gender-affirming care. JAMA Netw Open.

[5] Coleman E, Radix AE, Bouman WP (2022). Standards of care for the health of transgender and gender diverse people, version 8. Int J Transgend Health.

[6] Hembree WC, Cohen-Kettenis PT, Gooren L (2017). Endocrine treatment of gender-dysphoric/gender-incongruent persons: an Endocrine Society clinical practice guideline. J Clin Endocrinol Metab.

[7] Khan MHR, Alam MI, Hossain A (2025). Determinants of inequities in access to healthcare among transgender people in Bangladesh. Discov Soc Sci Health.

[8] Ministry of Social Welfare, Government of the People's Republic of Bangladesh (2014). Notification No. Sha.Ka.Ma./Karma-1 Sha./Hijra-15/2013-40, dated 09 Magh 1420 / 22 January 2014: recognition of the hijra population of Bangladesh as hijra gender.

[9] Abdullah MA, Basharat Z, Kamal B, Sattar NY, Hassan ZF, Jan AD, Shafqat A (2012). Is social exclusion pushing the Pakistani hijras (transgenders) towards commercial sex work? A qualitative study. BMC Int Health Hum Rights.

[10] Hossain A (2017). The paradox of recognition: hijra, third gender and sexual rights in Bangladesh. Cult Health Sex.

[11] Veeder TA, Leo RJ (2017). Male genital self-mutilation: a systematic review of psychiatric disorders and psychosocial factors. Gen Hosp Psychiatry.

[12] Miller IW, Norman WH, Bishop SB, Dow MG (1986). The modified scale for suicidal ideation: reliability and validity. J Consult Clin Psychol.

[13] Cipriani A, Hawton K, Stockton S, Geddes JR (2013). Lithium in the prevention of suicide in mood disorders: updated systematic review and meta-analysis. BMJ.

[14] Ryan C, Russell ST, Huebner D, Diaz R, Sanchez J (2010). Family acceptance in adolescence and the health of LGBT young adults. J Child Adolesc Psychiatr Nurs.

[15] Nuri NN, Sarker M, Ahmed HU, Hossain MD, Beiersmann C, Jahn A (2018). Pathways to care of patients with mental health problems in Bangladesh. Int J Ment Health Syst.

[16] (2026). "I want to live with my head held high": abuses in Bangladesh's legal recognition of hijras. http://2018https://www.hrw.org/report/2016/12/23/i-want-live-my-head-held-high/abuses-bangladeshs-legal-recognition-hijras.

[17] (2026). The Penal Code, 1860 (ACT NO. XLV OF 1860). http://bdlaws.minlaw.gov.bd/act-11/section-3233.html?hl=1.

